# Enhancing Bovine Embryo Development In Vitro Using Oil-in-Water Nanoemulsions as Specific Carriers for Essential Lipids

**DOI:** 10.3390/biotech13020019

**Published:** 2024-06-11

**Authors:** Daniel López Angulo, Rodrigo Vinicius Lourenço, Alessandra Bridi, Matheus Andrade Chaves, Juliano Coelho da Silveira, Paulo José do Amaral Sobral

**Affiliations:** 1 Department of Food Engineering, Faculty of Animal Science and Food Engineering, University of São Paulo, Pirassununga 13635-900, SP, Brazil; 2 Department of Veterinary Medicine, Faculty of Animal Science and Food Engineering, University of São Paulo, Pirassununga 13635-900, SP, Brazil; alessandra.brid@usp.br (A.B.);; 3 Food Research Center (FoRC), University of São Paulo, São Paulo 05508-000, SP, Brazil

**Keywords:** colloidal system, emulsion, lipid transport vehicle, enhancing embryonic development, bovine reproduction

## Abstract

Worldwide meat consumption and production have nearly quintupled in the last 60 years. In this context, research and the application of new technologies related to animal reproduction have evolved in an accelerated way. The objective of the present study was to apply nanoemulsions (NEs) as carriers of lipids to feed bovine embryos in culture media and verify their impact on the development of embryos produced *in vitro*. The NEs were characterized by particle size, polydispersity, size distribution, physical stability, morphology using atomic force microscopy (AFM), surface tension, density, pH, and rheological behavior. The NEs were prepared by the emulsification/evaporation technique. A central composite rotatable design (CCRD) was used to optimize the NE fabrication parameters. The three optimized formulations used in the embryo application showed an emulsion stability index (ESI) between 0.046 and 0.086, which reflects high stability. The mean droplet diameter analyzed by laser diffraction was approximately 70–80 nm, suggesting a possible transit across the embryonic zona pellucida with pores of an average 90 nm in diameter. AFM images clearly confirm the morphology of spherical droplets with a mean droplet diameter of less than 100 nm. The optimized formulations added during the higher embryonic genome activation phase in bovine embryos enhanced early embryonic development.

## 1. Introduction

The increasing population and food consumption are placing unprecedented demands on agriculture (livestock and crops) and natural resources. By 2050, the world’s population is expected to increase to around nine billion people [1]. Worldwide meat consumption and production nearly quintupled from 1961 to 2019 [2]. In this context, in the last decade, research and the application of new technologies related to animal reproduction have evolved in an accelerated way, resulting in the development of techniques that increase the reproductive capacity of bovine livestock. The techniques that have received most attention are artificial insemination, superovulation, in vitro fertilization, embryo culture, transfer and freezing, sexing of semen and embryos, and, more recently, embryo cloning [1]. Assisted reproduction plays a crucial role in animal husbandry, providing means to enhance reproductive efficiency and the genetic quality of livestock. However, one of the fundamental challenges faced by researchers and professionals in this field is ensuring the quality of embryos produced in vitro [3]. Embryo quality, primarily measured by the development rate to the blastocyst stage, is a vital indicator of in vitro fertilization success and subsequent pregnancy rates.

In this context, the lipid profile of a bovine embryo plays a crucial role in its quality and viability, as it infers the following: (a) Cell membrane integrity, because lipids are the main components of cell membranes. A healthy lipid profile becomes essential to maintaining the structural integrity of embryonic cells. Also, intact cell membranes are necessary for fundamental processes such as the regulation of nutrient and metabolite transport during embryonic development [4]; (b) the production of energy, since lipids can provide an efficient source of energy for embryonic cells after metabolization. Also, during embryonic development, especially when the embryo is progressing through rapid cell division stages, an adequate energy source is crucial for embryo viability [5]; (c) cell signaling and development, since certain lipids, such as phospholipids, can play important roles on signaling pathways that affect embryonic development, cell differentiation, and survival [6]; and (d) energy reserves, as embryos from mammals can produce and accumulate essential lipids that are used during the implantation phase, mostly when the embryo attaches and begins to develop in the maternal uterus [7].

Given this scenario, lipid supplementation involves the addition of specific sources of lipids to the culture medium, resulting in an enhancement of the embryo’s lipid profile and increased development. However, excessive or unbalanced lipid concentrations in the culture medium can be deleterious to embryos. High levels of lipids, particularly saturated fatty acids (SFAs), can lead to oxidative stress, impaired embryo development, and compromised quality. Lipid droplets within the embryo, known as lipid accumulation, can also be associated with poor embryo viability [8]. It is known that excessive lipid accumulation can occur in bovine embryos after in vitro production, probably due to the high absorption of lipids contained in lipoproteins from fetal calf serum, resulting in enhanced synthesis and accumulation of triacylglycerol (TAG) [9]. The buildup of TAGs and free fatty acids (FFAs) in embryos can harm mitochondrial and endoplasmic reticulum functions, leading to decreased developmental competence [10]. Efforts to mitigate the adverse impact of lipid accumulation involve adjusting the composition of culture media, minimizing or eliminating fetal calf serum (FCS), or incorporating lipolytic chemicals. Nonetheless, the outcomes have not yielded definitive conclusions [11].

To overcome these drawbacks, we considered that lipid nanocarriers could provide lipids of interest to embryo cells. To achieve this, we initially modified the composition of the culture medium by incorporating nanoemulsions (NEs) made from raw materials that are less likely to produce TAGs or FFAs when dispersed in aqueous media. NEs emerge as significant candidates for this application due to their production simplicity [12]. These systems are basically composed of droplets dispersed in a continuum medium, appearing as either oil-in-water (O/W) or water-in-oil (W/O) systems, with particle sizes in the nanometer range, specifically with a mean droplet size lower than 200 nm [13,14]. These characteristics give NEs an exceptional ability to encapsulate and deliver bioactive substances, such as nutrients and growth factors, in a stable and controlled manner. Nanoemulsified systems, by the way, could also enhance the uptake of lipids by cells due to their small droplet size and stable structure, ensuring their efficient incorporation into cellular membranes and metabolic pathways [15,16,17]. The high specific surface area of NEs is the main reason for the high bioavailability of the incorporated bioactive components.

Therefore, the objective of the present study was to apply NEs as carriers of lipids to feed bovine embryos in culture media and verify their impact on the development of embryos produced in vitro via parthenogenetic activation. To understand the effect of NEs on embryos, we conducted an extensive characterization of the colloidal systems, including analyses of particle size, polydispersity, size distribution, concentration using nanoparticle tracking analysis, physical stability, microstructure using atomic force microscopy, surface tension, density, pH, and rheological behavior. The relationship between the nanoemulsion’s lipid ratio and the improvement in the number of potential embryos was meticulously examined by the cleavage rate and the blastocyst rate, which provided valuable insights into the potential of these nanostructures as delivery vehicles/supplements in embryonic environments.

In this research, we explored the intersection of nanotechnology with reproductive biology, aiming not only to advance our understanding of the possible uses of nanoemulsions but also to provide perspectives for enhancing assisted reproduction techniques. By comprehending their influence on embryo quality, we hope to make significant contributions to the field, paving the way for innovative strategies for improving bovine reproduction and, consequently, sustainable and efficient livestock production.

Several nanostructured systems have been studied as a means of delivering active components in molecular biology and biomedicine studies [18,19,20,21,22,23]. Nevertheless, to the authors’ knowledge, no studies on the application of nanoemulsions in embryonic feeding were found in the literature.

## 2. Materials and Methods

### 2.1. Materials

Soybean L-α-phosphatidylcholine, type II-S, with a choline content of 14–29%, cholesterol (≥99%), and lecithin from Lipoid GmbH (300 M, Ludwigshafen, Germany) were procured from Sigma-Aldrich (St. Louis, MO, USA). Analytical-grade chloroform was purchased from Labsynth (Diadema, SP, Brazil). Distilled water from a Milli-Q purification system (Millipore, Billerica, MA, USA) was used as the aqueous phase during nanoemulsion production. For in vitro embryo production, all chemicals and reagents were obtained from Sigma-Aldrich (St. Louis, MO, USA) unless otherwise stated.

### 2.2. Production of Nanoemulsions

Two experiments were conducted in order to produce the oil-in-water NEs. In the initial experiment, the oily phase consisted solely of soybean phosphatidylcholine and lecithin. In the second experiment, NEs were produced using the optimal conditions identified in the first experiment, but a second compound of interest, cholesterol, was also introduced into the oily phase at 0.5 and 1% (*w*/*v*). The incorporation of cholesterol in the emulsion formulation was thought to provide structural, metabolic, and signaling support to the developing embryos [24].

### 2.3. Experimental Design

The first experiment aimed to establish the best nanoemulsion production conditions. A central composite rotational design (CCRD) was employed for this purpose [25]. Briefly, a complete 23 factorial design was performed, involving 6 axial points and 3 repetitions at the central point, resulting in a total of 17 runs. Data analysis was therefore performed using Statistica v.14.0 for Windows (TIBCO Software, Palo Alto, CA, USA). Dependent variables included the mean droplet diameter and the emulsion stability index (ESI), while the oil-to-water (O/W) ratio, the microfluidizer pressure (P), and the concentration of lecithin (CL) were set as independent variables. All variables and levels are summarized in Table 1. The experimental design matrix of coded values is presented in the Supplementary Data (Table S1).

### 2.4. Preparation of Nanoemulsions by the Emulsification/Evaporation Method

Nanoemulsions were prepared using the emulsification/evaporation method [26]. Initially, the oil phase, comprising phosphatidylcholine, lecithin, and chloroform, was stirred for 30 min under an extraction hood. Subsequently, the solution was slowly poured into the aqueous phase (see Table 1) using an Ultra-Turrax^®^ IKA T25 homogenizer (Labotechnik, Staufen, Germany) at 8000 rpm for 5 min to obtain a primary emulsion. This step was performed over an ice–water bath to maintain the temperature. Following this, the primary emulsion underwent further homogenization with a microfluidizer (M-110Y, Microfluidics, Westwood, MA, USA) through three cycles. Finally, the nanoemulsion was processed in a rotary evaporator (TE211, Tecnal, Piracicaba, Brazil) under a reduced pressure of 0.25 atm at 50 °C for 1 h to eliminate residual chloroform. Samples were then stored at 5 °C until analyzed.

### 2.5. Characterization of Nanoemulsions

#### 2.5.1. Droplet Size and Polydispersity Index (Span)

The nanoemulsion was analyzed using a laser-diffraction particle-size-distribution analyzer (Horiba, LA-950V2, Northampton, UK) equipped with an optical system featuring a 650 nm laser diode and a 405 nm light-emitting diode (LED), alongside silicon photodiode detectors. Prior to measurement, each sample underwent 1 min of ultrasonic treatment. The mean droplet diameter of the emulsions was expressed as surface-weighted mean diameters (*D*_3,2_) calculated using Equation (1).

(1) $$D_{3,2}=\frac{\sum n_{i}D_{i}^{3}}{\sum n_{i}D_{i}^{2}}$$

where *n_i_* and *D_i_* represent the number and diameter of the droplet population *i*, respectively.

Also, the distribution width of droplet size, known as the polydispersity index (PDI/Span), was calculated using Equation (2).

(2) $$Span=\frac{\left[d\left(v,90\right)-d\left(v,10\right)\right]}{d(v,50)}$$

where *d*(*v*, 10), *d*(*v*, 50), and *d*(*v*, 90) are diameters at 10%, 50%, and 90% cumulative volume, respectively.

#### 2.5.2. Physical Stability

The emulsion stability index (ESI) was determined using a multi-sample analytical centrifuge (LUMiSizer, LUM GmbH, Berlin, Germany) [25]. The applied operating parameters were as follows: the emulsion sample volume was 1.8 mL, the centrifugal force was set at 2325× *g*, the time was 400 s, the interval time was 10 s, and the temperature was maintained at 25 °C. ESI values range from 0 to 1, with a value closer to 1 indicating a high incidence of destabilization phenomena and a value closer to 0 indicates good stability of the nanoemulsion over time.

### 2.6. Characterization of NEs Produced under the Best Conditions

#### 2.6.1. Size Distribution and Droplet Concentration of Nanoemulsions

The nanoemulsions were examined for particle size and droplet concentration using nanoparticle tracking analysis (NTA). For this analysis, a 1:10,000 dilution in ultrapure water was used. Each sample was recorded in five 30 s videos using a scientific complementary metal–oxide–semiconductor (sCMOS) camera set at level 13 and maintained at a controlled temperature of 38.5 °C. The captured images were processed using NanoSight NTA 3.4 Analytical Software (NTA 3.4 Build 3.4.003; Malvern, PA, USA) with a threshold level of 5, providing data on particle size distribution and concentration for each sample.

#### 2.6.2. Morphology

To analyze the morphology of the nanoemulsion, 3D and phase images were obtained using an Atomic Force Microscope (AFM NT-MDT, Moscow, Russia) in height retrace mode and phase retrace mode, respectively. The NE samples were diluted tenfold with ultrapure water, and 10 μL of the diluted emulsion was deposited and dried on a flat mica sheet for microstructure observation. The scanning probe operated at a rate of 1 Hz with a drive frequency of 336 kHz on the mica sheet.

#### 2.6.3. Surface Tension

Surface tension was measured at 25 °C using a force tensiometer (Attension Sigma 702, Espoo, Finland). The measurements were performed in triplicate, and the standard uncertainty was estimated to be 1 Mn·m^−1^. The sample was placed inside a glass vessel, and a platinum Du Nouÿ ring was submerged in the sample until a higher tension was registered.

#### 2.6.4. Density and pH

The density of the nanoemulsions was determined at room temperature (20 °C) using a Gay-Lussac pycnometer. The design of the pycnometer allowed air bubbles to rise and concentrate in the neck, which were expelled when the self-flushing plug was placed. Prior to measurement, the pycnometer was calibrated, cleaned with detergent, rinsed with distilled water, followed by acetone, and then dried gently. The pH of all NEs was measured using a pHmeter (3505, Jenway, London, UK).

#### 2.6.5. Rheological Behavior

The flow behavior of the NEs was analyzed at 25 ºC using a rheometer (AR-2000, TA Instruments, New Castle, DE, USA) equipped with a double-walled concentric cylinder geometry (inner radius = 16 mm; outer radius = 17.5 mm; height = 53 mm; and gap = 2000 μm). Flow curves were obtained for strain rates from 0.01 to 200 s^−1^ in three cycles, involving both increase and decrease, with each ramp lasting 2 min.

#### 2.6.6. Application of NEs in In Vitro Embryo Production

In vitro embryo production was conducted following the method described by Sangalli et al. [27], with slight modifications. Briefly, ovaries were sourced from a local slaughterhouse and promptly transported to the laboratory in a saline solution (0.9% NaCl). Ovarian follicles with diameters ranging from 3 to 6 mm were aspirated to obtain the cumulus–oocyte complexes (COCs). The COCs displaying compact cumulus and homogeneous cytoplasm were carefully chosen and placed in in vitro maturation (IVM) medium. The IVM medium consisted of TCM199 culture medium (GIBCO BRL, Grand Island, NY, USA) supplemented with 50 μg/mL hCG (Vetercor^®^, Intervet, DE, USA), 1 μg/mL FSH (Vetoquinol, Fort Worth, TX, USA), 50 μg/mL gentamicin sulfate, 0.2 mM sodium pyruvate, and 10% fetal bovine serum (GIBCO BRL, Grand Island, NY, USA). The IVM process was performed for 22–24 h in a controlled environment with a humidified atmosphere of 5% CO_2_ in air at 38.5 °C. After IVM, matured oocytes had their cumulus cells removed. The denuded oocytes were washed with TCM199 236 containing 25 mM HEPES (GIBCO, Grand Island, NY, USA) and artificially activated by incubation with 5 μM ionomycin for 5 min. Following ionomycin activation, oocytes were washed three times in H199 with 30 mg/mL BSA, 2 mL/mL pyruvate, and 5 mL/mL gentamicin, and then washed three times and transferred into droplets of synthetic oviduct fluid (SOF) supplemented with 5 mg/mL BSA, 2.5% fetal bovine serum, 0.2 mM pyruvate, 10 mg/mL gentamicin, and 2 mM 6-dimethylaminopurine (DMAP) for 3 h culture at 38.5 °C and 5% CO_2_ in air.

Following parthenogenetic activation, the presumptive zygotes underwent a thorough wash using TCM199 medium modified with Hepes, supplemented with 22 µg/mL sodium pyruvate, 50 μg/mL gentamicin, and 0.1% BSA (bovine serum albumin). These zygotes were then carefully placed in a 4-well culture plate, with approximately 40–50 structures per well, and covered with 500 µL of SOFaaci cultured medium. The SOFaaci medium was supplemented with 8 mg/mL BSA, 22 μg/mL sodium pyruvate, and 50 μg/mL gentamicin. Subsequently, the culture plate was incubated at 38.5 ºC in a precisely controlled atmosphere with 6% CO_2_, 5% O_2_, and 89% N_2_ throughout the developmental period until day 7.

The presumptive zygotes were allocated into the following treatment groups:

(1) Control (C; without nanoemulsion);
(2) F1 (nanoemulsion composed of 5.0% phosphatidylcholine plus 0.0% cholesterol);
(3) F2 (nanoemulsion composed of 4.0% phosphatidylcholine plus 1.0% cholesterol);
(4) F3 (nanoemulsion composed of 4.5% phosphatidylcholine plus 0.5% cholesterol).

The nanodroplet concentration used in the treatment of the presumptive zygotes was equivalent to 2.5% of the total EVs present in 1 mL of oviduct flushing, which corresponds to 2.2 × 10^9^ nanodroplets/mL of medium. Cleavage and blastocyst rates were evaluated on days 3 and 7, respectively. This animal experiment was approved by the University Animal Care Committee at FZEA/USP (CEUA protocol number 2632030822).

### 2.7. Statistical Analysis

The data obtained in the first experiment with nanoemulsion characterizations were analyzed as described in Section 2.3, and the data obtained in the second experiment with nanoemulsion characterizations and applications, as well as the cleavage and blastocyst rate, were compared using ANOVA. Means were compared by Tukey’s test. An alpha of 5% was considered for all analyses.

## 3. Results

### 3.1. Results of the Experimental Design

The mean droplet diameter of the NEs varied from 64.6 ± 0.6 to 138.0 ± 0.4 nm (Table 2). It is significant to note that all samples were within the nanometric range, a notable observation given the purpose of our work. In this context, NEs must exhibit a smaller size than that observed for the pores of the zona pellucida in embryos, which are approximately 90 nm in the initial stages of embryonic development, to efficiently deliver lipids into their interior [28]. Moreover, phosphatidylcholine plays a crucial role in cell membrane formation, renewal, and the regeneration of damaged cells [8]. Regarding the process itself, the reduction in droplet size may be attributed to the high homogenization pressure and the number of cycles used during microfluidization. This reduction is likely due to the shear force under turbulent conditions in the interaction chamber of the equipment, leading to the rupture of interfacial membranes [29].

**Figure 1:**
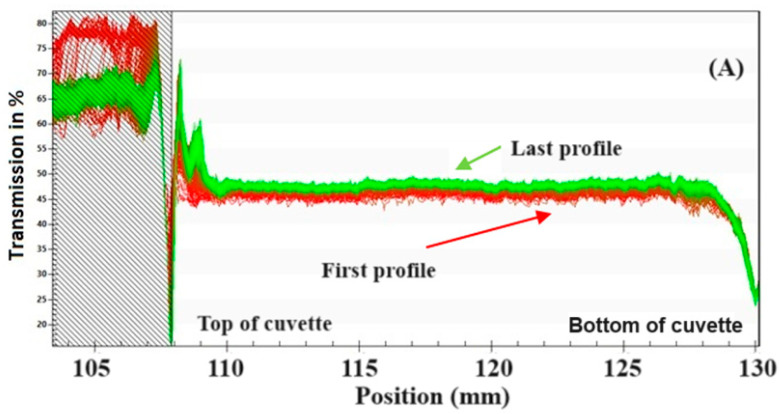
Examples of results of light transmission profile of a nanoemulsion in the first (run 4) experiment.

The physical stability of the NEs was studied with a multi-sample analytical photocentrifuge, which records, during centrifugation, the near-infrared light transmission over the total length of a cell containing the emulsion. Thus, it automatically determines the time dependence of the position of the interface of separated phases related to its stability. All NEs presented spectra of well-stabilized emulsions without the characteristics of creaming during running (Figure 1) [30]. The transmission profile of all runs was very similar, with no visible differences in the spectra. Therefore, it can be stated that all formulations would remain stable for approximately 3 months. The ESI varied between 0.055 ± 0.001 (run 5) and 0.146 ± 0.007 (run 11) (Table 2).

Overall, all samples presented a Span of around 0.2, indicating highly uniform size distributions, except for run 2, with a very high value (1.046 ± 0.006), explaining its high value for the mean droplet diameter. The overall value of Span showed that emulsions presented a very narrow volumetric distribution of size. This can help to explain the good stability of the NEs.

The results of the statistical analysis for the data presented in Table 2 have been provided in the Supplementary Materials file (Tables S2 and S3). The fitted coded models representing D_3,2_ (R^2^ = 0.68) and ESI (R^2^ = 0.32) are provided by Equations (3) and (4), respectively, as a function of X1 (P), X2 (O/W), and X3 (CL).

D_3,2_ = 111.6 − 0.52X_1_ + 10^−3^X_1_^2^ − 2.09X_2_ + 0.04X_2_^2^ + 35.25X_3_ + 12.12X_3_^2^ + 0.02X_1_X_2_ − 0.18X_1_X_3_ − 1.8X_2_X_3_. (3)

ESI = 0.097 + 4.1 × 10^−4^X_1_ − 2.0 × 10^−6^X_1_^2^ − 1.0 × 10^−3^X_2_ + 9.7 × 10^−5^X_2_^2^ − 0.04X_3_ − 3 × 10^−3^X_3_^2^ − 2.5 × 10^−5^X_1_X_2_ + 6.0 × 10^−4^X_1_X_3_ − 5.0 × 10^−4^X_2_X_3_. (4)

**Figure 2:**
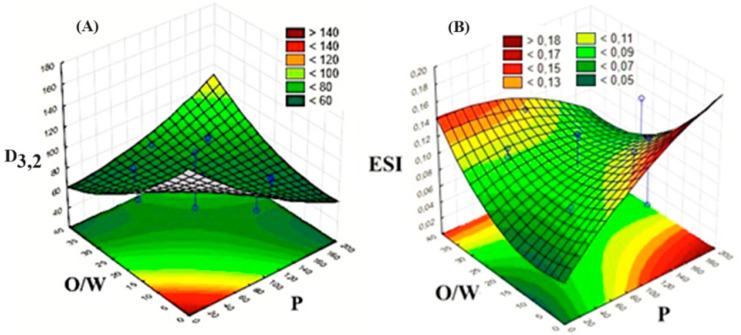
Mean droplet diameter (D_3,2_) (**A**) and emulsion stability index (**B**) as a function of O/W ratio and homogenization pressure within the central point of concentration of lecithin.

Equation (3) was used to build the response surface graphics (Figure 2A), allowing us to observe that pressure and lecithin concentration emerged as the most influential factors on D_3,2_ (*p* < 0.05). Higher pressure had a negative effect on low O/W values but a positive effect on high O/W values. Thus, lower D_3,2_ can be produced in a narrow zone between high (O/W)/low (P) and low (O/W)/high (P) (Figure 2A). This negative effect was similarly observed for the CL ratio. On the other hand, the concentration of lecithin did not exert a significant effect on D_3,2_ (*p* > 0.05). However, the pressure–O/W interaction was significant (*p* < 0.05).

Similarly, Equation (4) was used to generate Figure 2B. This response surface revealed that the effects of both the O/W ratio and the homogenization pressure also varied from one extremity to another. A lower ESI was obtained for low (O/W)/low (P) and high (O/W)/high (P) (Figure 2B). A similar trend was observed for ESI in relation to the CL.

After the analysis of the experimental design and according to the optimization zones of the response surface graphs, the best parameters considered were the following: p = 100 MPa, O/W = 20/80, and concentration of lecithin = 0.7% (Table 3).

### 3.2. Characterization of NEs for Embryo Culture Supplementation

Three formulations (F1, F2, F3) of NEs for embryo culture supplementation were produced using the formulations summarized in Table 3. Visually, no phase separations or agglomerations were observed in the nanoemulsions after three continuous cycles of microfluidization. The NEs were homogeneous, opaque, and white in color (see Figure S1 in the Supplementary Materials file).

#### 3.2.1. pH, Density, and Surface Tension of Nanoemulsions

All studied parameters showed remarkable similarity among all formulations (Table 4). pH values were close to those of pure water, without the effect of formulations. In this context, phospholipids such as phosphatidylcholine are typically not acidic or basic in nature; they do not release or consume hydrogen ions (H^+^) in a way that would significantly alter the pH. The same is true for cholesterol.

Regarding the density of the NEs, it is possible to observe that they closely resembled that of pure water, mostly due to the overall low lipid concentration. These results were interesting and beneficial for the application in the embryonic environment because water must have free movement into the embryos and, consequently, NEs too.

The superficial tension, which represents cohesive forces between the molecules of a liquid, was affected by the addition of cholesterol, which weakened the intermolecular forces facilitating the fluidity of NEs (Table 4). This means that cholesterol had an important surfactant effect in this system. This phenomenon could be attributed to the surfactant-like amphiphilic nature of cholesterol, since its structure consists of a sizable, nonpolar rigid planar fused-ring structure featuring a smooth side and a rough side, along with a brief flexible isooctyl tail and a small hydrophilic moiety in the form of a hydroxyl (−OH) group [31,32].

#### 3.2.2. Rheological behavior

**Figure 3:**
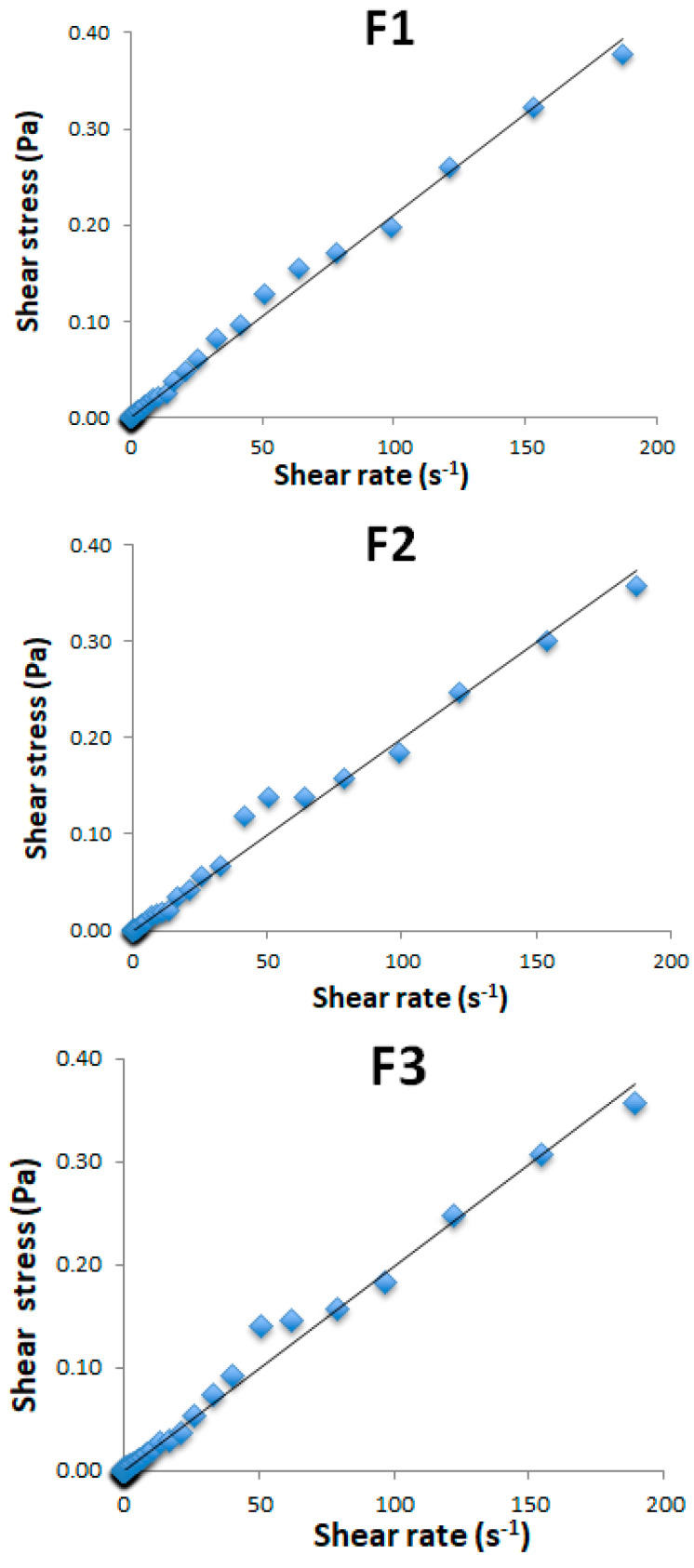
Flow curves for nanoemulsions produced under the best conditions.

The flow curves of the three NEs behaved as Newtonian fluids, where a linear relationship between shear stress and shear rate was observed (Figure 3).

Applying the Newtonian model (Equation (5)), it was possible to calculate the viscosity of these Nes, which stayed around 2 mPa·s (Table 4), close to that of pure water.

(5) $$\sigma =\mu \;\dot{\gamma }$$

where σ is the shear stress (mPa), $\dot{\gamma }$ is the shear rate (s^−1^), and µ is the viscosity (mPa·s) calculated by linear regression.

This result is important considering the migration across the embryo’s membrane, because this characteristic could facilitate the migration, mobility, and diffusion of the droplets carrying lipids during the early stages of embryonic development. Indeed, viscosity is related to the resistance to fluid flow [33].

#### 3.2.3. Morphology of NEs

**Figure 4:**
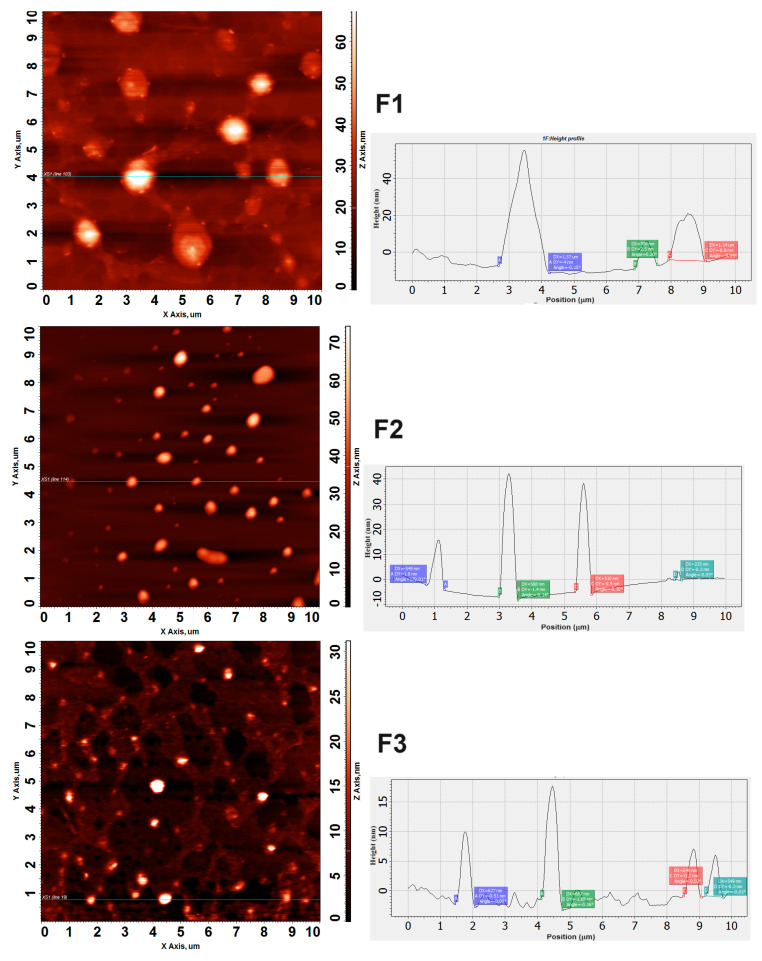
Two-dimensional AFM images with morphology (**left**) and height profile (**right**) of dry nanoemulsion F2.

The AFM results clearly confirmed a spherical morphology of oil droplets with sizes lower than 100 nm, as well as homogeneity in the droplet size distribution in the nanoemulsions (Figure 4). Several spherical structures were observed, as well as more elongated structures whose spherical shape could be deformed due to the loss of water that occurred on the support surface.

Moreover, the AFM results allowed us to observe the size profile of the structures (Figure 4). The horizontal line in the middle of the image shows a sweep of the cantilever across the mica surface, where the largest droplet reached a height of approximately 44 nm. This result is very interesting since it reveals the spherical morphology of the nanometric-sized structures, which is necessary for their application in the embryonic environment.

#### 3.2.4. Droplet Diameter Distribution

**Figure 5:**
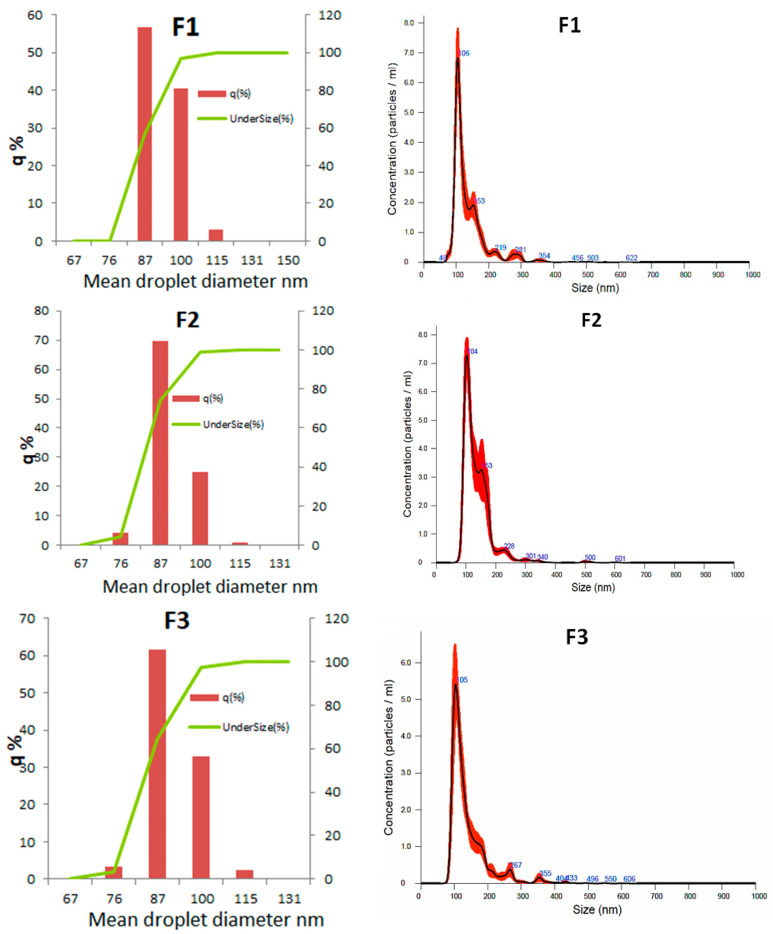
Drop size distribution by laser-diffraction particle analyzer (**left**) and by Nanosight (**right**).

All analyzed formulations presented a monomodal (Figure 5) droplet size distribution with a D_3,2_ ≈ 86nm and a Span ≈ 0.22 (Table 4). Therefore, it can be inferred that the three NEs had a narrow and monomodal size distribution [34].

It can be observed in Figure 5 that approximately 70% of the droplet population has a mean diameter lower or equal to 90 nm, which means, i.e., they are able to pass across the pores of the zona pellucida of embryos. The dynamic analysis using Nanosight revealed a high concentration of nanodroplets in the three formulations (2.8 × 10^14^ to 4.2 × 10^14^ droplets/mL), corroborating the nanometric size of the structures around ~100 nm on average (Figure 4).

#### 3.2.5. Physical stability of NEs

**Figure 6:**
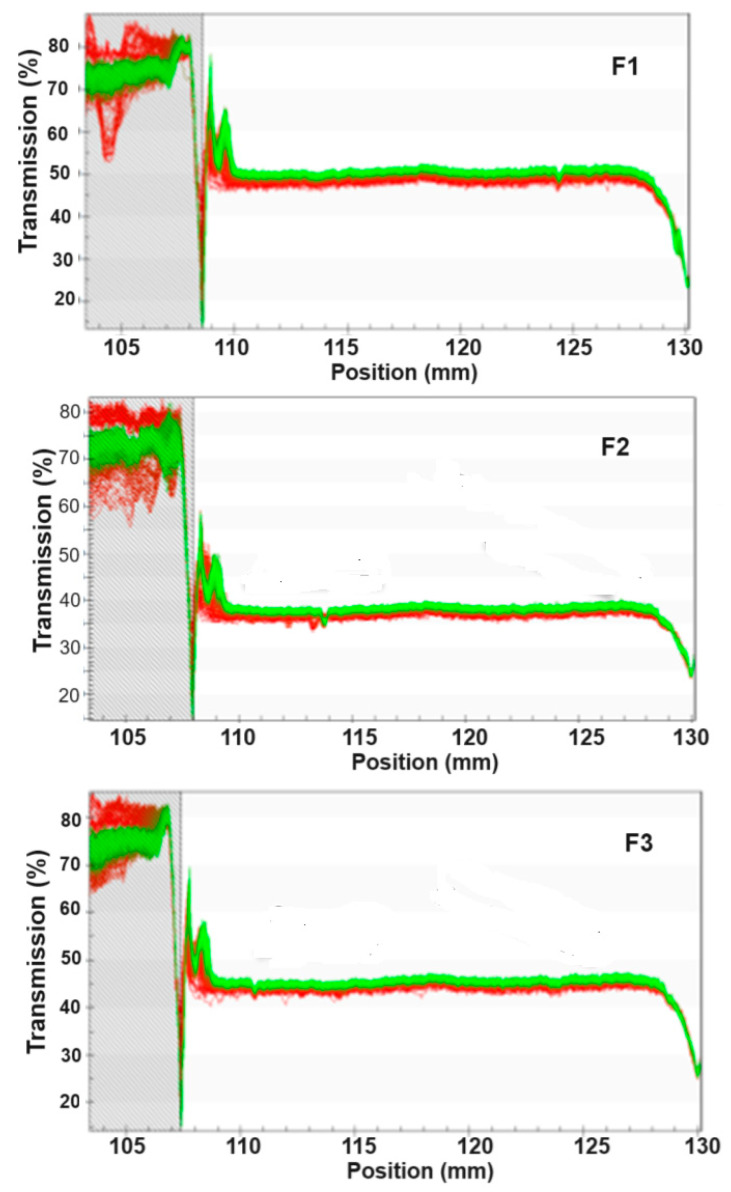
Light transmission profile of nanoemulsions produced under the best conditions.

Again, overall, all NEs were very stable, presenting light transmission profiles similar to those observed in the first experiments (Figure 6). No creaming was observed during or after the analysis. All ESI values were very low (<0.1), meaning that they were very stable (Table 4).

### 3.3. Applying Nanoemulsions for Bovine Embryonic Development

In order to evaluate if the nanoparticles present in nanoemulsions with different lipid compositions affect bovine early embryonic development, embryos were produced by parthenogenetic activation. Four biological replicates were performed, placing 204, 199, 201, and 203 COCs in the IVM for the Control, F1, F2, and F3, respectively.

**Figure 7:**
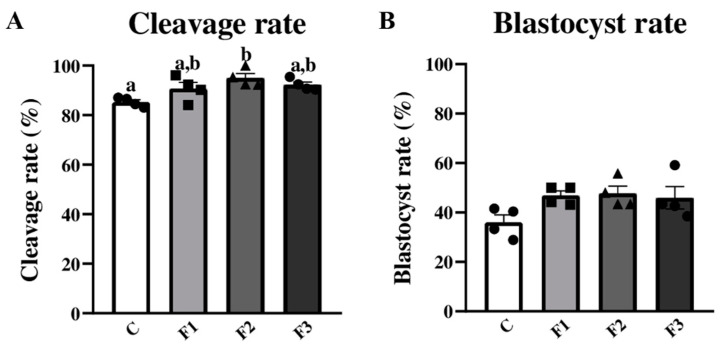
Cleavage rate (**A**) and blastocyst rate (**B**) were evaluated in bovine embryos treated or not with nanoemulsions with different lipid compositions. Solid bars represent the percentage of presumptive zygotes that cleaved or became blastocysts after parthenogenetic activation. Different lowercase letters represent significant differences (*p* ≤ 0.05).

The numbers of cleavages were 174, 181, 191, and 187 for the Control, F1, F2, and F3, respectively. The cleavage rate (Figure 7) was higher in the F2 group (95.02%) than in the Control group (85.29%; *p* = 0.0415). This result indicates that the F2 nanoemulsion, specifically during the higher embryonic genome activation phase in bovine embryos, enhances early embryonic development.

On the other hand, the numbers of embryos were 74, 93, 95, and 92 for the Control, F1, F2, and F3. Nevertheless, the blastocyst rate (Figure 7) was not different among the groups (*p* = 0.0929). It is worth mentioning that there was an increase of approximately ten percentual points in the blastocyst production rate in the F1, F2, and F3 groups when compared to the Control group. Therefore, taken collectively, these results provide strong evidence that nanoemulsions formulated with different lipid compositions, as well as the concentration employed in this study, do not exert any detrimental effects on early bovine embryonic development.

Assisted reproduction plays a crucial role in animal husbandry, providing means to enhance reproductive efficiency and the genetic quality of livestock, with positive consequences for the environment and the food industry as well. An increase in reproductive efficiency can culminate in a reduction in non-pregnant animals, which leads to an increase in animal protein production using less fresh water [35]. Genetic gain can contribute to the reduction in greenhouse emissions by animals due to a high food–energy conversion rate, leading to a reduction in the slaughtering age. According to Pickering et al. [36], the most sustainable way of reducing enteric CH_4_ emissions from ruminants is through genetic selection. On the other side, fast genetic gain can contribute to increasing meat quality. Indeed, the genetic variation that exists for meat quality is as large as that observed for most performance traits; thus, within a well-structured breeding program, rapid genetic gain for meat quality with direct consequences for the retail prices associated with the product could be possible [37]. Thus, our findings support the idea that nanoemulsions as carriers of lipids to feed bovine embryos in vitro are a possible tool to increase the success of the meat production chain. However, it should also be kept in mind that other researchers have focused in the production of emulsions intended for food application [25,30,34].

## 4. Conclusions

It was possible to obtain novel and very stable oil-in-water NEs, manufactured by a high-energy method, as specific transport vehicles for essential components. The optimized formulations by CCRD showed an ESI between 0.046 and 0.086, which reflects high stability with a very low incidence of destabilization phenomena. The average droplet diameter remained around 85 nm, and the AFM images clearly confirmed a spherical droplet morphology and the occurrence of droplets with an average size ≤ 100 nm.

These characteristics, together with the low viscosity of the NEs, allowed these colloidal systems to diffuse and migrate efficiently across the zona pellucida and transport the components of interest during embryonic development, highlighting that this new nanoemulsified system was not toxic and did not interfere with the aseptic conditions of the in vitro embryonic system.

An increase of approximately ten percentual points in the blastocyst production rate in the F1, F2, and F3 groups when compared to the Control group was observed. The F2 nanoemulsion, during the higher embryonic genome activation phase in bovine embryos, enhances early embryonic development. Finally, the NEs formulated with different lipid compositions, as well as the concentration employed in this study, do not exert any detrimental effects on early bovine embryonic development.

## Figures and Tables

**Table 1:** Values used in CCRD for NE production for the first experiment.

Variables	Code	Levels				
		−1.68	−1	0	1	1.68
Pressure (MPa)	X_1_	16	50	100	150	184
O/W ratio	X_2_	3/97	10/90	20/80	30/70	37/63
Concentration of Lecithin (% *w*/*w*)	X_3_	0.16	0.5	1.0	1.5	1.84

**Table 2:** Emulsion mean droplet diameter (D_3,2_) and stability index (ESI) of nanoemulsions.

Run	D_3,2_ (nm)	ESI
1	85.0 ± 0.2	0.087 ± 0.002
2	138.0 ± 0.4	0.058 ± 0.001
3	83.2 ± 0.5	0.099 ± 0.001
4	64.8 ± 0.4	0.106 ± 0.001
5	80.2 ± 0.1	0.055 ± 0.001
6	80.1 ± 0.9	0.131 ± 0.013
7	80.1 ± 0.0	0.063 ± 0.000
8	79.5 ± 0.4	0.077 ± 0.001
9	84.6 ± 0.7	0.052 ± 0.001
10	64.6 ± 0.6	0.139 ± 0.003
11	79.2 ± 0.1	0.146 ± 0.007
12	75.9 ± 0.1	0.124 ± 0.001
13	74.0 ± 0.6	0.127 ± 0.016
14	78.1 ± 0.0	0.081 ± 0.008
15	77.7 ± 0.3	0.128 ± 0.004
16	75.8 ± 0.1	0.078 ± 0.004
17	68.0 ± 9.0	0.061 ± 0.001

**Table 3:** Emulsion production conditions.

Treatment → Emulsion Production Conditions	F1	F2	F3
O/W ratio	20/80	20/80	20/80
Concentration of Lecitin (%)	0.7	0.7	0.7
Homogenization pressure (MPa)	100	100	100
Solvent (mL)	15	15	15
Phosphatidylcholine (%)	5.0	4.0	4.5
Cholesterol (%)	0	1	0.5

**Table 4:** Results of characterization of emulsions produced under the best conditions.

Treatment →	F1	F2	F3
pH	6.3 ± 0.0 ^a^	6.3 ± 0.1 ^a^	6.3 ± 0.0 ^a^
Density (kg/m^3^)	1.006 ± 0.0 ^a^	1.005 ± 0.0 ^a^	0.997 ± 0.0 ^a^
Surface tension (mN/m)	35.91 ± 2.67 ^a^	31.31 ± 1.82 ^b^	31.80 ± 0.50 ^b^
Viscosity (mPa.s)	2.1 ± 0.2 ^a^	2.0 ± 0.0 ^a^	2.0 ± 0.1 ^a^
Mean droplet diameter (nm)	86.4 ± 1.0 ^a^	83.8 ± 0.7 ^a^	86.0 ± 0.4 ^a^
Polidispersion index	0.224 ± 0.003 ^a^	0.214 ± 0.004 ^a^	0.228 ± 0.004 ^a^
Emulsion stability index	0.083 ± 0.003 ^a^	0.047 ± 0.001 ^b^	0.057 ± 0.004 ^b^

Different letters in the same row represent significant differences (*p* ≤ 0.05).

## Data Availability

The data that support the findings of this study are available from the corresponding author upon reasonable request.

## Supplementary Materials

The following supporting information can be downloaded at https://www.mdpi.com/article/10.3390/biotech13020019/s1, Figure S1: Macroscopic appearance of nanoemulsions; Table S1: Experimental design matrix of coded values; Table S2: Anova for mean droplet diameter (MDD); Table S3: Results of Anova for emulsion stability index (ESI).
