# Supplementary material for: Enhancing Bovine Embryo Development In Vitro Using Oil-in-Water Nanoemulsions as Specific Carriers for Essential Lipids
Source: BioTech (Basel). 2024 Jun 11;13(2):19. doi: 10.3390/biotech13020019 (PMC11201380; doi:10.3390/biotech13020019)
Supplement: Supplementary file 1 [file biotech-13-00019-s001.zip › biotech-2997705-supplementary.pdf]

Supplementary Material

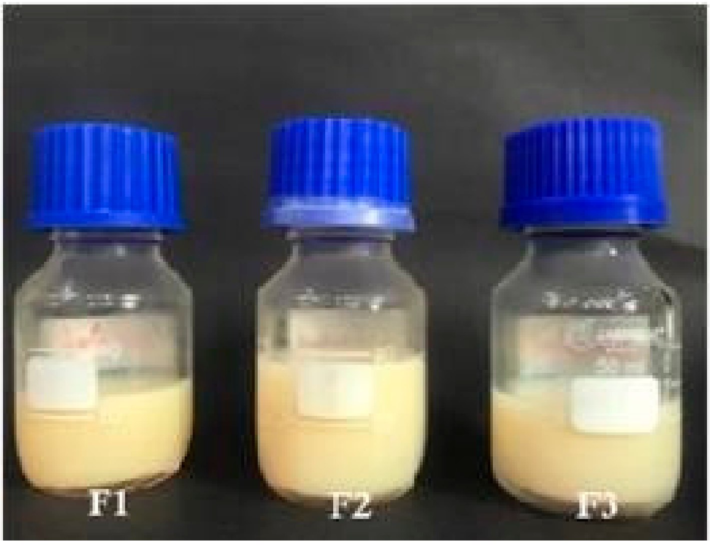

Figure S1. Macroscopic appearance of nanoemulsions.

Table S1. Experimental design matrix of coded values.

Run	P	CL	O/W ratio
1	-	-	-
2	+	-	-
3	-	+	-
4	+	+	-
5	-	-	+
6	+	-	+
7	-	+	+
8	+	+	+
9	- α	0	0
10	+ α	0	0
11	0 (100)	- α	0
12	0 (100)	+ α	0
13	0 (100)	0 (1.0)	- α
14	0 (100)	0 (1.0)	+ α
15	0 (100)	0 (1.0)	0 (20/80)
16	0 (100)	0 (1.0)	0 (20/80)
17	0 (100)	0 (1.0)	0 (20/80)

The values (-1), (0) and (1) are coded levels.

The matrix shown in table 1 was obtained by applying a Central Composite Rotatable Design (CCRD) with software Statistica 14.0, including 6 axis points and 3 repetitions in the central point, totaling 17 runs.

Table S2. Anova for mean droplet diameter (MDD).

	SS	df	MS	F	p
(1)P (L)	524,177	1	524,1773	20,17756	0,046156
P (Q)	71,775	1	71,7747	2,76288	0,238366
(2)OW (L)	482,149	1	482,1495	18,55975	0,049883
OW (Q)	143,801	1	143,8011	5,53545	0,142919
(3)CL (L)	122,202	1	122,2023	4,70403	0,162341
CL (Q)	103,540	1	103,5401	3,98565	0,183993
1L by 2L	693,036	1	693,0365	26,67758	0,035501
1L by 3L	154,880	1	154,8800	5,96191	0,134665
2L by 3L	645,123	1	645,1232	24,83322	0,037989
Lack of Fit	1226,996	5	245,3992	9,44634	0,098487
Pure Error	51,956	2	25,9782		
Total SS	4106,019	16			

Table S3. Results of Anova for emulsion stability index (ESI).

	SS	df	MS	F	p
(1)P (L)	0,00090	1	0,000908	0,698514	0,491226
P (Q)	0,00022	1	0,000223	0,171273	0,719142
(2)OW (L)	0,00006	1	0,000068	0,052238	0,840456
OW (Q)	0,00106	1	0,001060	0,815596	0,461789
(3)CL (L)	0,00000 1	1	0,000001	0,000942	0,978302
CL (Q)	0,00000 9	1	0,000009	0,007159	0,940277
1L by 2L	0,00125	1	0,001250	0,961538	0,430197
1L by 3L	0,00180	1	0,001800	1,384615	0,360398
2L by 3L	0,00005	1	0,000050	0,038462	0,862639
Lack of Fit	0,00956	5	0,001914	1,472061	0,451705
Pure Error	0,00260	2	0,001300		
Total SS	0,01802	16			

* L: lineal interaction and Q: quadratic interaction
